# Comparative Analysis of Corneal Parameters in Swept-Source Imaging between DMEK and UT-DSAEK Eyes

**DOI:** 10.3390/jcm10215119

**Published:** 2021-10-30

**Authors:** Anna Machalińska, Agnieszka Kuligowska, Bogna Kowalska, Krzysztof Safranow

**Affiliations:** 1 First Department of Ophthalmology, Pomeranian Medical University, 70-111 Szczecin, Poland; agnieszka.kaleta91@gmail.com (A.K.); bogna.kowalska96@gmail.com (B.K.); 2 Department of Biochemistry and Medical Chemistry, Pomeranian Medical University, 70-111 Szczecin, Poland; chrissaf@mp.pl

**Keywords:** UT-DSAEK, DMEK, keratometry, astigmatism, HOA

## Abstract

Background: The need to provide a comparative analysis of corneal parameter changes compared to their preoperative values between Descemet membrane endothelial keratoplasty (DMEK) and ultrathin Descemet stripping automated endothelial keratoplasty (UT-DSAEK) patients. Methods: The study included 24 eyes after UT-DSAEK and 24 eyes after DMEK. Visual acuity, endothelial cell count (ECC), central corneal thickness (CCT), mean keratometry (MK), mean astigmatism (MA), astigmatism asymmetry (AA) and higher-order aberrations (HOAs) were assessed at baseline and 1, 3, 6 and 12 months after the surgery. Results: From the 3rd month post operation, ECC was higher in the DMEK eyes than in the UT-DSAEK eyes (*p* = 0.01). In a bivariate analysis that was adjusted for age, DMEK was associated with a smaller decrease in posterior MK at the 1-month (β = −0.49, *p* = 0.002), 3-month (β = −0.50, *p* < 0.001), 6-month (β = −0.58, *p* < 0.001) and 12-month (β = −0.49, *p* < 0.001) follow-up visits. There were no significant differences in changes in anterior or combined surface MK throughout the observation period. Accordingly, no significant differences in changes in MA, AA or HOAs compared to the baseline values were identified between the eyes after DMEK and UT-DSAEK at any follow-up time point. Conclusions: UT-DSAEK seemed to be an easier and safer technique than DMEK while maintaining similar outcomes regarding irregular astigmatism and total keratometry values.

## 1. Introduction

The introduction of endothelial keratoplasty (EK) has revolutionized corneal transplantation over almost the past two decades. Descemet stripping automated endothelial keratoplasty (DSAEK), which is a procedure that involves the selective removal of the dysfunctional endothelium, followed by the transplantation of donor corneal endothelium, Descemet membrane (DM) and a portion of donor corneal stroma, replaced penetrating keratoplasty (PK). Accordingly, Descemet membrane endothelial keratoplasty (DMEK), comprising the selective transplantation of a donor button that is composed of endothelium and DM without the posterior stroma, replaced DSAEK as the most common type of corneal transplantation [1,2].

In 2013, Busin et al. pointed out that ultrathin Descemet stripping automated endothelial keratoplasty (UT-DSAEK) might be a procedure that shares the improved visual outcome and lower immunologic rejection rate of DMEK over DSAEK while minimizing all types of postoperative complications. It was also highlighted that, similar to DSAEK and unlike DMEK, UT-DSAEK can be performed for all types of eyes, even those with complicated anatomy or poor anterior chamber visualization [3].

There are only a few studies that compared DMEK and UT-DSAEK, and they mainly focused on visual acuity outcomes, contrast sensitivity, endothelial cell loss or complication rates [4,5,6,7,8,9,10]. Some studies also compared the patient-recorded outcome measures between both techniques [6,10,11,12]. Chamberlain et al. documented that DMEK had superior visual acuity results with similar complication rates compared with UT-DSAEK at 3, 6 and 12 months post surgery in patients with isolated endothelial dysfunction [9]. In contrast, reports from other groups provided evidence that DMEK and UT-DSAEK did not differ significantly in terms of visual acuity [4,6,8]. Mencucci et al. emphasized that DMEK and UT-DSAEK show no difference in postoperative best-corrected visual acuity (BCVA), although DMEK has a better performance in terms of contrast sensitivity and overall patient satisfaction [7].

Only a few studies have compared higher-order aberrations, astigmatism, corneal pachymetry and keratometry using either Schleimpfung cameras or tomographs with corneal topographers between eyes that have undergone either DMEK or UT-DSAEK. The concomitantly growing popularity of anterior segment optical coherent tomography (AS-OCT) has caused the use of this device in clinical practice to become increasingly frequent. To the best of our knowledge, to date, there have been no studies that compared corneal topographic parameters between eyes that underwent DMEK and UT-DSAEK in swept-source AS-OCT. Bearing in mind the above, our aim was to create a study to provide an analysis of corneal parameter changes in patients who underwent DMEK and UT-DSAEK using an AS-OCT device.

## 2. Materials and Methods

This study included 24 consecutive eyes that had undergone UT-DSAEK and 24 consecutive eyes that had undergone DMEK due to various causes of endothelial decompensation (Fuchs endothelial corneal dystrophy or pseudophakic bullous keratopathy). The subjects that were enrolled in the study were patients that were operated on at the First Ophthalmology Clinic in Szczecin in 2018-2020 and then followed up at 1, 3, 6 and 12 months after the surgery. All participants underwent a complete ophthalmologic examination, including the following: best-corrected distance visual acuity with Snellen charts (Remote-Controlled Chart Monitor, CC-100, Topcon, Tokyo, Japan), slit lamp biomicroscopy, IOP measurements and a detailed fundus examination after pupil mydriasis. Corneal quality parameters were measured with swept-source AS-OCT at each following visit. 

### 2.1. Donor Characteristics

Donor corneas were obtained with the multiorgan procurement method and in the dissecting room during autopsies. Corneoscleral buttons were hypothermically stored in Eusol-C medium (Alchimia, Ponte San Nicolò, Italy) at 2–6 °C at the West Pomeranian Eye Tissue Bank in Szczecin. The prestorage evaluation of the endothelium was performed using specular microscopy (Konan CellCheck EB-10; Konan Medical USA Inc., Irvine, CA, USA). All corneas had an endothelial cell count of at least 2800 cells/mm^2^.

### 2.2. Surgical Techniques

Each surgery was performed by the same surgeon. All subjects underwent prophylactic basal laser iridectomy (Optimis Fusion, Quantel Medical, Cournon-d’Auvergne, France) before endothelial keratoplasty to minimize the risk of postoperative pupillary block. Subjects with retinal diseases that significantly affected visual acuity were excluded from the study. All procedures were performed with peribulbar block.

UT-DSAEK grafts were prepared with the single-pass technique using a MORIA One Use microkeratome. The donor tissue was mounted on an artificial anterior chamber (AAC), which maintained a continuous intracameral pressure of 200 mmHg (Moria S.A., Antony, France). After ensuring adequate pressure in the AAC system, central corneal thickness measurements were taken using an ultrasound iPac Pachymeter (Reichert, Inc., Depew, NY, USA). An appropriate microkeratome head was chosen, and the cut was performed at a deliberately slow speed. The anterior cap was removed, and the residual stromal bed was again measured. The AAC was carefully disassembled, avoiding any trauma to the endothelium.

The donor’s rolled endothelial graft was inserted using a Busin glide through a 3.2 mm clear corneal incision (Moria S.A., Antony, France). After centering the graft, the anterior chamber was filled with air to allow for a perfect adherence of the donor flap to the receiving tissue.

The preparation of the DMEK graft and surgery was performed following the ‘no-touch’ technique, as described previously [13].

All patients were instructed to keep a supine position after the surgery till the control of the flap position was done on the first postoperative day. In the case of a pupillary block or ocular hypertension, topical mydriatics were administered, and if this was insufficient, a small quantity of air was released from the AC in the operating theatre. The postoperative treatment for both groups was a topical antibiotic given 4 times a day for 1 week and topical preservative-free dexamethasone sodium phosphate 8 times a day for the first month. The topical steroid was tapered down to one drop every other day and then discontinued over 1 year. The graft thickness in all UT-DSAEK eyes was below 100 µm (mean graft thickness at the 12-month follow-up visit was 66.09 ± 18.61 µm). 

### 2.3. AS-OCT Measurements

Both the corneal thickness and keratometry values were determined using a swept-source anterior segment OCT CASIA2 (Tomey, Nagoya, Japan). During the entire observation period, the CASIA2 was placed in the same room under the same lighting conditions. All measurements were taken by trained operators, who also held the subjects’ eyelids gently to avoid pressure on the globe. The scan was performed using the autoalignment function. The CASIA2 measurements were obtained with the corneal map mode of the anterior segment module from the OCT images using the built-in software and measurement tools provided by the manufacturers. The CASIA software automatically defined the intraocular structures and generated measurement values. The images were analyzed using built-in 2D analysis software that automatically calculated the measurements, along with the structural outlines and reference lines. The outline tracer was edited where needed.

Central corneal thickness (μm), mean keratometry values (D), astigmatism power (D) and axis (°), astigmatism asymmetry (D) and higher-order aberration power (D) were recorded and analyzed at different time points after surgery using the Fourier Analysis 3D/2D function. Measurements were read from both the anterior and posterior surfaces of the cornea, and the total values were taken into account. All parameters were assessed in 3 and 6 mm diameter optical zones (OZs). The image quality was assessed during the acquisition by the operator. Only measurements that were well-centered and with high-quality indexes were included in the study.

### 2.4. Statistical Analysis

Statistical analysis was conducted using Statistica software. Since most of the analyzed variables showed distributions that were significantly different from normal distributions (Shapiro–Wilk test, *p* < 0.05), non-parametric tests were used. The Wilcoxon signed-rank test was used to compare the preoperative and postoperative values. The Mann–Whitney U test was applied to analyze the differences between the groups. The correlations between the baseline variables and the corneal parameters were analyzed with Spearman’s rank correlation coefficient (Rs). Bivariate analysis, including surgical technique and patient’s age as independent variables, and change of a parameter relative to its baseline value as the dependent variable, was performed using a general linear model (GLM). Standardized beta values (β) were presented to show the strength of association between the surgical technique and the follow-up parameter changes. A *p*-value of less than 0.05 was considered to be statistically significant. The statistical power of the study at the 0.05 significance level with 24 subjects in each study group was sufficient to detect with 80% probability the real effect size corresponding to the difference between the groups equal to ±0.83 SD and associations between variables within groups with a real effect size corresponding to the correlation coefficient of ±0.54 [14].

## 3. Results

### 3.1. Characteristics of the Study Groups

Forty-eight eyes of 43 patients were assigned to DMEK (*n* = 24) or UT-DSAEK (*n* = 24). Table 1 provides the preoperative characteristics of the study groups. The study groups were matched for sex (*p* = 0.55) and the DMEK group was younger than the UT-DSAEK group (*p* = 0.01). At the baseline, there were no significant differences between the groups in the values of corneal topographic parameters, i.e., the mean keratometry (MK) values, astigmatism power and asymmetry (AA) and the values of higher-order aberrations (HOAs). Accordingly, the groups did not differ in preoperative central corneal thickness measurements. 

Three patients in the UT-DSAEK group were lost at the 12-month follow-up point. No graft failures or rejections were observed in this study. In four eyes after UT-DSAEK and three eyes after DMEK surgery, a postoperative graft detachment was observed. All of them were recorded in the first 24 h after surgery, with one exception: one DMEK graft detachment took place one week after surgery. In this case, the patient admitted to not following the postoperative recommendations. All detached grafts were successfully attached due to intracameral SF_6_ or air injection. In four eyes after DMEK, there was a need to partially remove SF_6_ from the anterior chamber in the first 24 h after surgery due to the elevated intraocular pressure (IOP). No further complications in those eyes were observed.

### 3.2. Postoperative Changes in BCVA and ECC in DMEK and UT-DSAEK Eyes

The mean baseline BCVA was 0.22 in the DMEK arm and 0.11 in the UT-DSAEK arm (*p* = 0.01). Up to 6 months post operation, the BCVA improved in both treatment groups to a similar extent from baseline. After adjusting for age, the change in BCVA did not differ significantly between DMEK and UT-DSAEK patients at the 1-month follow-up visit (β = −0.25, *p* = 0.12), 3-month follow-up visit (β = −0.04, *p* = 0.76) or 6-month follow-up visit (β = −0.22, *p* = 0.06). In contrast, at the 12-month follow-up visit, the postoperative increase in the BCVA was higher in the DMEK group (median = 1.00) than in the UT-DSAEK group (median = 0.6, *p* < 0.001). This difference remained significant in the multivariate analysis that was performed using a GLM after an adjustment for age (β = −0.37, *p* = 0.006).

Regarding the ECC, we observed no differences in the donor endothelial cell count (median = 3045.50 cells/mm^2^ in the DMEK group and median = 3140 cells/mm^2^ in the UT-DSAEK group, *p* = 0.26). From 3 months post operation, the endothelial cell density was higher in the DMEK eyes than in the UT-DSAEK eyes (median = 1470.50 cells/mm^2^ and median = 1156 cells/mm^2^, *p* = 0.009, respectively, at the 3-month follow-up visit; median = 1384 cells/mm^2^ and median = 1146 cells/mm^2^, *p* = 0.005, respectively, at the 6-month follow-up visit; median = 1304.5 cells/mm^2^ and median = 1113 cells/mm^2^, *p* = 0.07, respectively, at the 12-month follow-up visit).

### 3.3. Postoperative Analysis of AS-OCT Corneal Parameters in DMEK and UT-DSAEK Eyes Compared to Baseline

First, we analyzed the changes in corneal surface parameters compared to baseline recordings for the DMEK and UT-DSAEK groups separately. Table 2 and Table 3 provide the corneal topography values of the postoperative pachymetry, keratometry, astigmatism and aberration parameters that were obtained for the DMEK and UT-DSAEK groups of eyes, respectively. A significant decrease in CCT compared to baseline recordings was observed at all follow-up time points in both the DMEK and UT-DSAEK groups.

At 1, 3, 6 and 12 months after DMEK surgery, there were no significant differences in anterior surface mean keratometry values compared to the baseline in the 3.0 and 6.0 mm diameter optical zones (6.0 mm diameter—data not shown). For the posterior surface, MK values were lower at all follow-up time points than preoperative values. Interestingly, for the combined corneal surface, there were no differences in MK values between those recorded at 6 and 12 months post operation and the baseline. Contrary to the DMEK group, in the UT-DSEAK group, we observed a significant decrease in MK recordings for both the anterior and posterior corneal surfaces, as well as for total corneas in both the 3.0 and 6.0 mm diameter OZs as early as 1 month post operation (6.0 mm diameter—data not shown). Finally, the combined surface MK values were significantly lower at 12 months post operation than the preoperative values (mean difference = −1.42 ± 1.87 D for the 3 mm OZ).

Regarding the astigmatism power, in the DMEK group, we observed a significant decrease for the posterior and total corneas from 3 months post operation and the anterior corneal surface from 6 months post operation. In contrast, in the UT-DSAEK eyes, the astigmatism power remained unchanged until 6 months post operation and decreased for the anterior and total corneas only at 12 months post operation. The magnitude of the posterior astigmatism in the UT-DSAEK eyes remained unchanged throughout the observation period. Interestingly, we found no significant changes in the astigmatism axis at 1, 3, 6 and 12 months after surgery compared to the baseline in either the DMEK or UT-DSAEK group.

When analyzing the irregular corneal astigmatism with the asymmetry of astigmatic components and HOAs, we observed similar patterns of decrease in both the UT-DSAEK and DMEK subgroups. At 1, 3, 6 and 12 months after surgery in both investigated groups, the values of the astigmatism asymmetry and HOAs decreased significantly compared to the preoperative values.

### 3.4. Comparative Analysis of Postoperative Corneal Surface Parameters between DMEK and UT-DSAEK Eyes

**Figure 1:**
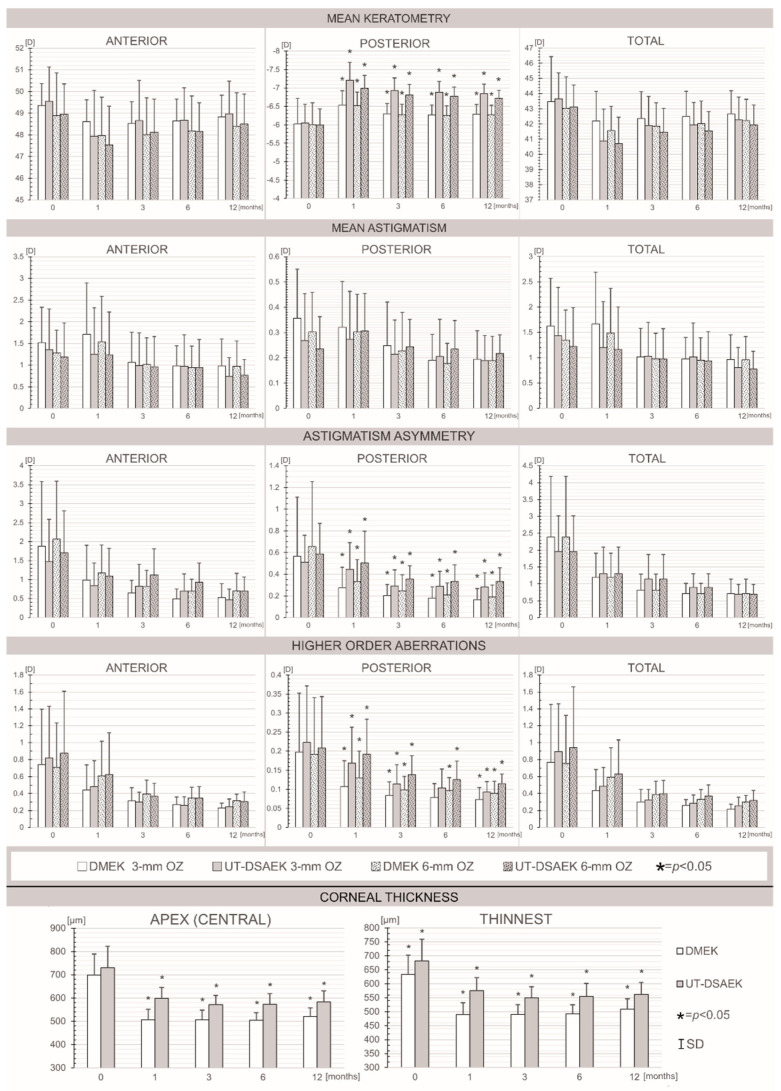
Bar graphs showing the pre- and postoperative mean values of the corneal parameters, which were evaluated using swept-source AS-OCT: mean keratometry, mean astigmatism, astigmatism asymmetry, higher-order aberrations and corneal thickness at the baseline and 1, 3, 6 and 12 months after Descemet membrane endothelial keratoplasty (DMEK) versus ultrathin Descemet stripping automated endothelial keratoplasty (UT-DSAEK) measured in 3 and 6 mm optical zones (OZs). * *p* < 0.05.

Figure 1 shows the postoperative corneal parameters, as evaluated using swept-source AS-OCT, including pachymetry, keratometry, astigmatism magnitude and asymmetry, as well as HOAs in the different corneal zones after UT-DSAEK and DMEK.

Throughout the observation period, we found no differences in the values of any parameters for the anterior and total corneas between the DMEK and UT-DSAEK eyes. Importantly, the values of the posterior mean keratometry and the magnitudes of the back astigmatism asymmetry and HOAs were significantly lower in the DMEK group than in the UT-DSAEK group at all follow-up time points. No differences in astigmatism power for either the anterior or posterior region or the total cornea were observed at any time point between the analyzed groups of eyes.

Subsequently, we compared the changes in corneal surface parameters at postoperative follow-up time points relative to the preoperative values between the DMEK and UT-DSAEK groups. Regarding the pachymetry, we found no differences in the CCT changes at the 1-, 3- and 6-month follow-up visits between the study groups. Exclusively, at the 12-month follow-up visit, the decrease in the CCT was larger in the DMEK eyes (β = +0.32, *p* = 0.04) in the multivariate analysis performed using a GLM after an adjustment for age. A multivariate analysis of keratometry changes in the DMEK and UT-DSAEK eyes, after an adjustment for age, revealed that DMEK was an independent variable that was associated with a smaller decrease in posterior mean keratometry at the 1-month (β = −0.49, *p* = 0.002), 3-month (β = −0.50, *p* < 0.001), 6-month (β = −0.58, *p* < 0.001) and 12-month (β = −0.49, *p* < 0.001) follow-up visits in both the 3 and 6 mm optical zones. There were no significant differences in changes in anterior surface or combined surface MK between the UT-DSAEK and DMEK groups throughout the observation period. Regarding the astigmatism power, there were no significant differences in the anterior, posterior surface or combined surface astigmatism changes between the UT-DSAEK and DMEK groups, with one exception: posterior astigmatism decreases at the 6- and 12-month follow-up visits were larger in the DMEK group (β = +0.40, *p* = 0.01) in the multivariate analysis that was performed using a GLM after an adjustment for age. Accordingly, no significant differences in changes in astigmatism asymmetry and HOAs compared to baseline values were identified between the eyes from the DMEK and UT-DSAEK groups at any follow-up time point.

### 3.5. Correlations between BCVA and Corneal Parameters at Subsequent Follow-Up Points

Next, we investigated the potential relationships between the corneal parameters and BCVA. We found that the baseline BCVA determined the postoperative corneal thickness at the 12-month follow-up in both the UT-DSAEK and DMEK groups. Accordingly, we observed a positive correlation between the preoperative BCVA and changes in the CCT at the 1-month (Rs = +0.59, *p* = 0.003), 3-month (Rs = +0.54, *p* = 0.006), 6-month (Rs = +0.57, *p* = 0.004) and 12-month (Rs = +0.52, *p* = 0.01) follow-up visits in the DMEK eyes. Analogous correlations were observed in the UT-DSAEK eyes at 1 month (Rs = +0.59, *p* = 0.01), 3 months (Rs = +0.58, *p* = 0.006), 6 months (Rs = +0.46, *p* = 0.04) and 12 months (Rs = +0.33, *p* = 0.17) post operation.

To identify the corneal surface aberrations that most affected the BCVA at subsequent postoperative visits, we investigated the associations between the BCVA and topographic characteristics of the cornea in both the DMEK and UT-DSAEK groups (Table 4). In the UT-DSAEK eyes, we observed a negative correlation between the BCVA and astigmatism power at 3, 6 and 12 months post operation. Accordingly, the BCVA was negatively correlated with the astigmatism asymmetry throughout the observation period. No such correlations were found in the DMEK group.

### 3.6. Correlations between CCT Change and Changes in Corneal Parameters Compared to Baseline Values

We also analyzed the potential associations between the changes in selected corneal parameters at selected time points post operation. We found that decreases in the magnitudes of the astigmatism, HOA and AA values for the total and posterior cornea throughout the observation period, as well as the anterior corneal surface at 6 and 12 months post operation (Table 5), were positively associated with a decrease in the CCT in the DMEK eyes. Interestingly, in the UT-DSAEK eyes, an analogous relationship was observed only between changes in the CCT and HOAs.

## 4. Discussion

In recent years, endothelial corneal transplantation has evolved to become the method of choice for the treatment of endothelial damage. Although DMEK seems to offer better and faster visual and refractive results, controversy still exists regarding whether it is a better technique than UT-DSAEK and NT-DSAEK, which are easier techniques and have similar outcomes to DMEK.

The results of this study provided a comparison of a wide range of topographic outcomes between DMEK and UT-DSAEK during a 1-year follow-up period. Compared with the UT-DSAEK results, lower mean posterior keratometry, lower posterior astigmatism, lower posterior astigmatism asymmetry and fewer posterior corneal HOAs were found after DMEK. Nevertheless, total corneal parameters were comparable after both procedures.

The results of this study indicated significant differences in posterior surface MK values between the keratoplasty techniques, which occurred after surgery. Compared to DMEK, we noted the steeper posterior corneal curvature in the UT-DSAEK group. This finding is consistent with the results of previous studies. Both Torras-Sanvicens et al. and Goldich et al. analyzed the mean anterior and posterior keratometry values and, similarly to us, found significant differences in the posterior keratometry in favor of DMEK [6,15]. Importantly, the combined surface UT-DSAEK MK values were significantly lower at 12 months post operation than before the operation by −1.42 ± 1.87 D. In contrast, after DMEK surgery, we found no significant differences in the anterior and combined surface MK values throughout the observation period compared to the baseline.

On the other hand, hyperopic outcomes tend to occur after posterior lamellar corneal transplantation. This hyperopic shift is very relevant after DSAEK and is described in the literature as ranging from 0.7 to 1.5 D [16]. Regarding UT-DSAEK, Busin et al. reported a significant hyperopic shift of 0.78 ± 0.59 D at the 1-year follow-up [3]. On the other hand, In their multicenter, prospective, double-masked, randomized, controlled clinical trial comparing DSAEK and UT-DSAEK, Dickman et al. observed a comparable hyperopic shift after both procedures [17]. Similarly, Dunker et al. showed no difference in hyperopic shift after the UT-DSAEK and DMEK surgery techniques [8].

In this study, we demonstrated a significant decrease in astigmatism power in the eyes after DMEK surgery that occurred from 3 months after surgery in the anterior, posterior and total corneas. In contrast, this effect was significantly prolonged in the anterior and total corneas and occurred only after 12 months post operation. We found no changes in posterior astigmatism throughout the observation period in the UT-DSAEK group. This allowed us to assume that the visual rehabilitation of patients after DMEK was shorter than that after UT-DSAEK. Indeed, data provided by Mencucci et al. lead to a similar assumption that postoperative recovery after DMEK might be quicker than that after UT-DSAEK [7]. Our results are also in line with those from the randomized controlled DETECT trial, which documented that DMEK was associated with a postoperative shift in posterior corneal astigmatism but not UT-DSAEK [18]. 

HOAs may increase after endothelial keratoplasty, which would significantly reduce visual acuity and influence vision quality. Throughout the observation period, we found no differences in the astigmatism asymmetry or HOA values for the anterior and total corneas between the DMEK and UT-DSAEK eyes. Importantly, the posterior absolute values of those parameters were significantly lower in the DMEK group than in the UT-DSAEK group at all follow-up time points. It is noteworthy that previous analyses that compared the changes in HOAs between DMEK and UT-DSAEK showed inconclusive results. In a recent study performed in 2020, Duggan et al. evaluated the outcomes of DMEK vs. UT-DSAEK patients and found that DMEK induced less posterior corneal HOAs than UT-DSAEK. The authors reported that posterior corneal HOA actually increased significantly in the UT-DSAEK group from the baseline to 3 months after surgery and remained significantly higher at 6 and 12 months. The HOAs of the anterior corneal surface in their sample did not differ significantly between DMEK and UT-DSAEK. These results are only partially in line with ours since we documented a similar pattern of decrease in the astigmatism asymmetry and the HOAs of the anterior, posterior and total corneas in both the DMEK and UT-DSAEK groups. Likewise, Mencucci et al. conducted a similar investigation and found that total and posterior corneal HOAs, posterior astigmatism and total coma were significantly lower after DMEK than after UT-DSAEK in both the 4 and 6 mm optical zones [7]. On the other hand, Torras-Sanvicens et al. did not observe statistically significant differences in total, anterior or posterior HOAs 12 months after surgery [6], which is in line with the results of other groups [19]. Altogether, it should be highlighted that the published data differ in terms of the operating techniques, corneal topographer device, definitions of UT-DSAEK grafts below 100 μm [8,9,20] or below 130 μm [3,7] and selection of study groups used, which makes it impossible to draw definite conclusions. It is worth mentioning that none of the previous studies compared the postoperative corneal curvature and topography parameters with their baseline values recorded preoperatively. To overcome this limitation, our study, for the first time, compared the changes in astigmatism asymmetry and HOAs compared to the baseline values between DMEK and UT-DSAEK eyes. We documented no significant differences between the two groups in the relative variations in any high-order aberration parameter at any follow-up time point. Our data seemed to represent an objective value in the comparative analysis between the UT-DSAEK and DMEK eyes. This approach eliminates the potential influence of the baseline corneal status on postoperative results between the study groups.

Bearing in mind that the presence of HOAs might affect the vision-related quality of life, several research groups have analyzed the objective and subjective visual quality and contrast sensitivity after both procedures. In their cross-sectional, comparative, and observational case series, Torras-Sanvicens et al. conducted an extended and comprehensive analysis that included objective visual quality variables, contrast sensitivity and subjective patient satisfaction, e.g., subjective quality of the surgical technique, level of comfort in the postoperative period, recovery time and preferred eye. The authors found no significant differences in any of the parameters studied between UT-DSAEK and DMEK eyes [6]. Accordingly, other research groups, e.g., Dukner et al., Ang et al. and Dunbar et al., reported no differences in vision-related quality of life between both procedures [10,11,12]. Interestingly, we observed that a decrease in the magnitudes of HOA values throughout the observation time was positively associated with a decrease in CCT. This strongly indicated that corneal thickness influenced the changes in corneal curvature and indicated a dynamic balance between corneal parameters due to the healing process within corneal tissue. Accordingly, postoperative visual acuity was significantly correlated with topographic characteristics of the cornea (the higher the aberrations, the lower the visual acuity was) in the UT-DSAEK eyes at subsequent postoperative visits. Similar observations were recorded by other study groups [7]. Importantly, our study seems to be the first to suggest that baseline vision acuity determines postoperative corneal thickness at the 12-month follow-up in both the DSAEK and DMEK groups. Further evidence is needed to establish causative links between changes in BCVA, central corneal thickness and corneal topographic outcomes after DMEK and UT-DSAEK surgery.

The results of our study clearly demonstrated that ECC loss after DMEK was less than that after UT-DSAEK. These results are comparable with those published by other research groups [6]. Conversely, Mencucci et al. [7] found no difference in ECC count between DMEK and UT-DSAEK eyes. Similar results were provided by Dunker et al. [8]. This observation is in line with those of several comparative DMEK and DSAEK studies that documented that endothelial cell loss was similar for both procedures [17,21,22]. The probable cause of such imbalances was, according to the authors, the increased handling of DMEK tissue during surgery. With regard to all the studies mentioned above, we can assume that endothelial cell loss after surgery is an individual quality that depends on the surgical technique, the experience of the operator and donor tissue preparatory procedures.

In conclusion, to the best of our knowledge, this is the first case series that compared changes in corneal topographic parameters with their baseline values between UT-DSAEK and DMEK groups at the 12-month follow-up. Accordingly, this might also be the first study in which the values of corneal topographic parameters in DMEK and UT-DSAEK eyes were studied using CASIA2 swept-source anterior segment OCT. Although DMEK showed a faster recovery during follow-up, along with lower pachymetry and a flatter posterior keratometry than UT DSAEK, UT-DSAEK seemed to be an easier and safer technique with similar outcomes regarding irregular corneal astigmatism with asymmetry of astigmatic components and HOAs.

## Figures and Tables

**Table 1:** Preoperative characteristics of the study groups (mean values ± SD). Statistically significant *p*-values are shown in bold.

			UT-DSAEK	DMEK	*p*
Recipient age (y)			74.96 ± 10.43	66.25 ± 11.23	**0.01**
Recipient sex (m/f)			9/13	5/16	0.55
Indications for the surgery (FECD/PBK)			14/10	21/3	n/a
Baseline BCVA			0.11 ± 0.12	0.22 ± 0.15	**0.01**
Donor graft ECC (cells/mm^2^)			3129.96 ± 242.48	3057.83 ± 317.73	0.26
Baseline CCT (μm)			730.04 ± 92.67	698.92 ± 89.61	0.20
Baseline mean keratometry (D)	Anterior	3 mm OZ	49.55 ± 1.58	49.36 ± 2.72	1.00
		6 mm OZ	48.96 ± 1.39	48.89 ± 1.97	0.78
	Posterior	3 mm OZ	−6.05 ± 0.51	−6.02 ± 0.69	0.90
		6 mm OZ	−5.99 ± 0.43	−6.00 ± 0.59	0.97
	Total	3 mm OZ	43.66 ± 1.71	43.48 ± 2.94	1.00
		6 mm OZ	43.12 ± 1.44	43.04 ± 2.06	0.89
Baseline mean astigmatism (D)	Anterior	3 mm OZ	1.35 ± 0.95	1.52 ± 0.81	0.34
		6 mm OZ	1.19 ± 0.78	1.28 ± 0.52	0.29
	Posterior	3 mm OZ	0.27 ± 0.18	0.36 ± 0.19	0.10
		6 mm OZ	0.24 ± 0.13	0.30 ± 0.16	0.14
	Total	3 mm OZ	1.43 ± 0.96	1.63 ± 0.94	0.47
		6 mm OZ	1.22 ± 0.77	1.35 ± 0.59	0.28
Baseline astigmatism asymmetry (D)	Anterior	3 mm OZ	1.48 ± 1.10	1.88 ± 1.97	0.65
		6 mm OZ	1.71 ± 1.10	2.06 ± 1.80	0.73
	Posterior	3 mm OZ	0.51 ± 0.25	0.56 ± 0.55	0.60
		6 mm OZ	0.59 ± 0.28	0.66 ± 0.60	0.62
	Total	3 mm OZ	1.72 ± 1.10	2.10 ± 1.97	0.97
		6 mm OZ	1.95 ± 1.06	2.38 ± 1.80	0.66
Baseline higher-order aberrations (D)	Anterior	3 mm OZ	0.82 ± 0.61	0.74 ± 0.65	0.40
		6 mm OZ	0.88 ± 0.73	0.70 ± 0.52	0.32
	Posterior	3 mm OZ	0.22 ± 0.15	0.19 ± 0.15	0.40
		6 mm OZ	0.21 ± 0.13	0.19 ± 0.15	0.46
	Total	3 mm OZ	0.89 ± 0.57	0.77 ± 0.68	0.11
		6 mm OZ	0.94 ± 0.72	0.75 ± 0.57	0.17

UT-DSAEK—ultrathin Descemet membrane automated endothelial keratoplasty, DMEK—Descemet membrane endothelial keratoplasty, FECD—Fuchs endothelial corneal dystrophy, PBK—pseudophakic bullous keratopathy, BCVA—best-corrected visual acuity, ECC—endothelial cell count, CCT—central corneal thickness, n/a—not applicable.

**Table 2:** Changes in the central corneal thickness (CCT), mean keratometry, astigmatism magnitude, astigmatism asymmetry and higher-order aberration power compared to the baseline in the 3 mm corneal optical zone after Descemet membrane endothelial keratoplasty (DMEK). Statistically significant values are shown in bold.

		Baseline	1 Month		3 Months		6 Months		12 Months	
		Median (IQR)	Median (IQR)	*p*	Median (IQR)	*p*	Median (IQR)	*p*	Median (IQR)	*p*
Corneal thickness (μm)	Apex	680.5 (129)	503 (69)	**<0.001**	498 (56)	**<0.001**	507 (51)	**<0.001**	517.5 (42.5)	**<0.001**
	Thinnest	618.5 (70)	490 (69)	**<0.001**	486.5 (44)	**<0.001**	493 (42)	**<0.001**	508 (43)	**<0.001**
Mean keratometry (D)	Anterior	49.96 (4.96)	48.34 (2.85)	0.50	48.11 (2.90)	0.36	49.02 (2.68)	0.54	49.26 (2.68)	0.36
	Posterior	−6.06 (0.89)	−6.47 (0.40)	**<0.001**	−6.37 (0.35)	**0.02**	−6.32 (0.41)	**0.06**	−6.34 (0.31)	**0.03**
	Total	43.88 (4.46)	42.07 (2.18)	0.24	41.92 (2.29)	0.19	42.76 (2.21)	0.31	42.92 (2.31)	0.17
Mean astigmatism (D)	Anterior	1.47 (1.01)	1.58 (1.28)	0.9	0.93 (0.83)	0.06	0.92 (0.68)	**0.02**	0.88 (0.90)	**0.03**
	Posterior	0.30 (0.30)	0.28 (0.20)	0.44	0.22 (0.13)	**0.03**	0.19 (0.13)	**0.001**	0.18 (0.14)	**0.001**
	Total	1.31 (1.57)	1.63 (1.15)	0.79	0.89 (0.825)	**0.03**	0.93 (0.6)	**0.02**	0.835 (0.51)	**0.02**
Astigmatism asymmetry (D)	Anterior	1.11 (1.79)	1.00 (0.59)	**0.01**	0.57 (0.44)	**0.004**	0.50 (0.29)	**<0.001**	0.49 (0.30)	**<0.001**
	Posterior	0.43 (0.54)	0.24 (0.23)	**0.01**	0.18 (0.09)	**0.002**	0.15 (0.1)	**<0.001**	0.13 (0.09)	**<0.001**
	Total	1.41 (1.69)	0.78 (0.66)	**0.001**	0.62 (0.42)	**0.002**	0.51 (0.32)	**<0.001**	0.55 (0.39)	**<0.001**
Higher-order aberrations (D)	Anterior	0.48 (0.98)	0.34 (0.37)	0.08	0.28 (0.19)	**0.007**	0.28 (0.15)	**0.006**	0.22 (0.10)	**<0.001**
	Posterior	0.18 (0.16)	0.09 (0.05)	**0.002**	0.08 (0.06)	**0.003**	0.08 (0.06)	**0.003**	0.07 (0.05)	**0.004**
	Total	0.46 (0.85)	0.37 (0.33)	**0.046**	0.27 (0.15)	**0.001**	0.27 (0.1)	**<0.001**	0.21 (0.08)	**<0.001**

**Table 3:** Changes in the central corneal thickness (CCT), mean keratometry, astigmatism magnitude, astigmatism asymmetry and higher-order aberration power compared to the baseline in the 3 mm corneal optical zone after ultrathin Descemet stripping automated endothelial keratoplasty (UT-DSAEK). Statistically significant values are shown in bold.

		Baseline	1 Month		3 Months		6 Months		12 Months	
		Median (IQR)	Median (IQR)	*p*	Median (IQR)	*p*	Median (IQR)	*p*	Median (IQR)	*p*
Corneal thickness (μm)	Apex	696 (100)	600.5 (50)	**<0.001**	567 (63)	**<0.001**	577 (71)	**<0.001**	590 (62)	**<0.001**
	Thinnest	656 (84)	572 (49)	**<0.001**	544 (58)	**<0.001**	553 (69)	**<0.001**	558 (53)	**<0.001**
Mean keratometry (D)	Anterior	49.31 (2.32)	48.38 (2.14)	**0.008**	49.09 (2.15)	**0.06**	48.85 (1.38)	**0.02**	48.86 (1.64)	0.13
	Posterior	−6.02 (0.97)	−7.12 (0.86)	**<0.001**	−6.94 (0.27)	**<0.001**	−6.89 (0.27)	**<0.001**	−6.85 (0.24)	**<0.001**
	Total	43.53 (2.16)	41.37 (2.21)	**<0.001**	42.22 (2.16)	**0.002**	42.01 (1.43)	**<0.001**	42.14 (1.29)	**0.004**
Mean astigmatism (D)	Anterior	1.28 (1.46)	1 (1.23)	0.57	0.83 (0.65)	0.11	0.82 (0.66)	0.12	0.61 (0.65)	**0.017**
	Posterior	0.23 (0.24)	0.23 (0.23)	0.98	0.21 (0.2)	0.4	0.17 (0.21)	0.24	0.18 (0.11)	0.24
	Total	1.32 (1.41)	0.79 (1.32)	0.42	0.9 (0.84)	0.11	0.87 (0.73)	0.13	0.78 (0.4)	**0.02**
Astigmatism asymmetry (D)	Anterior	1.02 (1.03)	0.63 (0.59)	**0.01**	0.69 (0.93)	**0.002**	0.52 (0.67)	**<0.001**	0.49 (0.41)	**<0.001**
	Posterior	0.5 (0.22)	0.36 (0.25)	0.54	0.3 (0.32)	**0.001**	0.29 (0.17)	**<0.001**	0.25 (0.21)	**<0.001**
	Total	1.38 (1.4)	0.87 (0.69)	**0.02**	0.74 (0.66)	**0.001**	0.62 (0.52)	**<0.001**	0.41 (0.23)	**<0.001**
Higher-order aberrations (D)	Anterior	0.63 (0.71)	0.41 (0.25)	**0.007**	0.27 (0.09)	**<0.001**	0.22 (0.13)	**<0.001**	0.23 (0.06)	**<0.001**
	Posterior	0.19 (0.18)	0.14 (0.13)	0.43	0.1 (0.08)	**0.002**	0.09 (0.05)	**<0.001**	0.09 (0.04)	**0.002**
	Total	0.73 (0.69)	0.45 (0.16)	**0.001**	0.31 (0.18)	**<0.001**	0.26 (0.11)	**<0.001**	0.24 (0.1)	**<0.001**

**Table 4:** The correlations between the best-corrected visual acuity (BCVA) and the magnitudes of the keratometry, astigmatism, astigmatism asymmetry and higher-order aberrations that were obtained in the 3 mm optical zones in the eyes after Descemet membrane endothelial keratoplasty (DMEK) and ultrathin Descemet stripping automated endothelial keratoplasty (UT-DSAEK) procedures. Correlations were calculated for the baseline state and then for 4 consecutive time points, i.e., 1, 3, 6 and 12 months, after the surgery. Statistically significant values are shown in bold.

Correlation		Baseline		1 Month		3 Months		6 Months		12 Months	
		UT- DSAEK	DMEK	UT- DSAEK	DMEK	UT- DSAEK	DMEK	UT- DSAEK	DMEK	UT- DSAEK	DMEK
Mean keratometry	Anterior	+0.13	+0.17	−0.10	+0.03	+0.11	−0.29	−0.08	+0.01	+0.41	+0.22
	Posterior	+0.32	+0.24	+0.36	+0.19	+0.34	**+0.52**	+0.31	+0.26	**+0.54**	+0.04
	Total	+0.09	+0.18	−0.01	+0.02	+0.15	−0.22	−0.03	+0.09	+0.45	+0.32
Mean astigmatism	Anterior	**−0.45**	+0.11	−0.45	−0.08	**−0.43**	−0.22	**−0.53**	−0.29	−0.39	−0.03
	Posterior	−0.17	−0.22	−0.17	−0.09	+0.02	+0.08	+0.16	−0.03	−0.27	−0.04
	Total	−0.41	+0.05	−0.41	−0.12	−0.39	−0.27	**−0.50**	−0.29	**−0.54**	−0.20
Astigmatism asymmetry	Anterior	**−0.52**	−0.19	−0.26	+0.18	**−0.54**	−0.29	−0.40	−0.39	**−0.58**	−0.36
	Posterior	+0.44	−0.10	**−0.58**	−0.33	−0.35	−0.21	−0.34	−0.34	−0.03	−0.34
	Total	−0.31	−0.19	−0.39	+0.18	**−0.43**	−0.17	−0.14	−0.15	+0.06	+0.03
Higher-order aberrations	Anterior	**−0.46**	−0.03	+0.01	+0.06	−0.33	−0.32	−0.32	−0.10	−0.14	−0.04
	Posterior	−0.28	−0.26	−0.26	−0.02	−0.09	−0.05	−0.15	**−0.49**	−0.16	−0.14
	Total	−0.41	−0.08	−0.11	−0.05	−0.31	−0.30	−0.42	−0.01	−0.29	−0.04

**Table 5:** The correlations between the central corneal thickness (CCT) change and changes in the magnitudes of the keratometry, astigmatism, astigmatism asymmetry and higher-order aberrations that were obtained in 3 mm optical zones (OZs) in the eyes after Descemet membrane endothelial keratoplasty (DMEK) and ultrathin Descemet stripping automated endothelial keratoplasty (UT-DSAEK) procedures. Correlations were calculated for 4 consecutive time points, i.e., 1, 3, 6 and 12 months after the surgery. Statistically significant values are shown in bold.

Correlation		1 Month		3 Months		6 Months		12 Months	
		UT- DSAEK	DMEK	UT- DSAEK	DMEK	UT- DSAEK	DMEK	UT- DSAEK	DMEK
Mean keratometry change	Anterior	+0.43	−0.002	+0.01	−0.05	+0.06	−0.03	+0.05	+0.17
	Posterior	−0.15	−0.01	−0.11	−0.08	−0.02	−0.09	+0.14	−0.003
	Total	+0.38	+0.04	+0.02	−0.05	+0.04	−0.04	+0.07	+0.16
Mean astigmatism change	Anterior	−0.12	+0.28	−0.15	**+0.45**	−0.03	**+0.47**	+0.15	**+0.65**
	Posterior	−0.01	**+0.64**	−0.05	**+0.54**	−0.0005	**+0.48**	+0.06	**+0.64**
	Total	−0.08	+0.39	−0.07	**+0.46**	−0.03	**+0.52**	+0.22	**+0.72**
Astigmatism asymmetry change	Anterior	+0.36	+0.15	+0.33	+0.07	+0.11	−0.22	+0.22	−0.05
	Posterior	+0.01	+0.28	+0.13	**+0.47**	+0.11	+0.38	+0.11	**+0.50**
	Total	+0.20	**+0.67**	+0.18	**+0.60**	+0.31	**+0.57**	+0.22	**+0.65**
Higher-order aberrations change	Anterior	−0.05	**+0.44**	+0.26	**+0.47**	+0.27	**+0.50**	+0.18	**+0.58**
	Posterior	**+0.52**	**+0.75**	**+0.74**	**+0.75**	**+0.76**	**+0.61**	**+0.77**	**+0.72**
	Total	+0.20	**+0.45**	+0.38	**+0.54**	**+0.44**	**+0.64**	+0.18	**+0.66**

## Data Availability

The data that were used to support the findings of this study are available from the corresponding author upon request.
